# Wrappers for Medical Journals

**Published:** 1871-05

**Authors:** 


Dr. Butler, of the *Philadelphia Medical and Surgical Reporter*, puts his journal in a pink wrapper as soon as a new subscription is due, and when payment is made the brown wrapper is resumed. If the pink should not succeed in a reasonable time, why not try black?—*Pacific Medical Journal.*

